# Mucin-Related Molecular Responses of Bronchial Epithelial Cells in Rats Infected with the Nematode *Nippostrongylus brasiliensis*

**DOI:** 10.5402/2013/804585

**Published:** 2013-03-16

**Authors:** Koichi Soga, Minoru Yamada, Yuji Naito, Toshikazu Yoshikawa, Naoki Arizono

**Affiliations:** ^1^Department of Molecular Gastroenterology and Hepatology, Kyoto Prefectural University of Medicine, Kawaramachi-Hirokoji, Kyoto 602-8566, Japan; ^2^Department of Medical Zoology, Kyoto Prefectural University of Medicine, Kyoto 602-8566, Japan

## Abstract

Although mucins are essential for the protection of internal epithelial surfaces, molecular responses involving mucin production and secretion in response to various infectious agents in the airway have not been fully elucidated. The present study analysed airway goblet cell mucins in rats infected with the nematode *Nippostrongylus brasiliensis*, which migrates to the lungs shortly after infection. Goblet cell hyperplasia occurred in the bronchial epithelium 3–10 days after infection. The high iron diamine-alcian blue staining combined with neuraminidase treatment showed that sialomucin is the major mucin in hyperplastic goblet cells. Immunohistochemical studies demonstrated that goblet cell mucins were immunoreactive with both the major airway mucin core peptide, Muc5AC, and the major intestinal mucin core peptide Muc2. Reverse transcription real-time PCR studies demonstrated upregulation of gene transcription levels of Muc5AC, Muc2, the sialyltransferase St3gal4, and the resistin-like molecule beta (Retnlb) in the lungs. These results showed that nematode infection induces airway epithelial responses characterised by the production of sialomucin with Muc5AC and Muc2 core peptides. These mucins, as well as Retnlb, might have important roles in the protection of mucosa from migrating nematodes in the airway.

## 1. Introduction

Parasitic gastrointestinal nematodes are one of the most commonly acquired infections in the world, affecting approximately one-quarter of the human population [1]. Parasitic helminths also place a considerable constraint upon the livestock industry, representing a major economic burden; one recent estimate suggested that *£*1.7 billion is spent annually on their control [2]. Although they cause relatively little mortality, infections result in high levels of morbidity that can result in developmental consequences in infected children [3] and cause significant economic loss in infected animals [2]. The morbidity induced by these infections is thought to be mediated through a combination of effects on both the nutrition and immunological responses of the host [3].

Certain intestinal nematodes, such as *Ascaris*, hookworms, and *Strongyloides,* migrate to the lungs before homing to their final habitat, the small intestine. Although some patients develop lung migration-associated symptoms, such as asthmatic cough, peripheral blood eosinophilia, and urticaria, which are collectively classified as pulmonary infiltration with eosinophilia (PIE) syndrome, the majority of patients are asymptomatic when the larval nematodes pass through the airways, suggesting that a highly effective protective barrier might be produced between the larvae and airway mucosa. Little is known about the molecular aspects of the responses such as the species of mucins and nonmucin secretory peptides produced in the bronchial epithelium in response to nematode infection, although infection with nematodes such as *Nippostrongylus brasiliensis* and *Strongyloides venezuelensis* had been reported to result in goblet cell hyperplasia in the bronchial epithelium [4, 5].

Airway mucins are pivotal to maintaining epithelial homeostasis, and mucin has been regarded as an ancient defence mechanism used by primitive organisms to create a barrier between themselves and noxious environmental stimuli. Mucins with highly O-linked glycoproteins are expressed either at the cell surface, or as secreted molecules to form a protective gel [6, 7]. In normal conditions, mucins protect and lubricate the epithelial surface, and trap particles including bacteria and viruses for mucociliary clearance [8]. Two major secretory mucins include mucin core peptide (Muc) 2 and Muc5AC. Muc2 is prominent mucins expressed in normal intestinal mucosa [9], while Muc5AC is normally expressed in airway epithelial cells [10–12]. Studies on mucins in human airway epithelial cells infected with the bacterial pathogen *Pseudomonas aeruginosa* showed increased expression of Muc2 and Muc5AC [13–15]. Mucins are classified into neutral and acidic subtypes; the latter are further distinguished by sulfated (sulfomucin) or nonsulfated (sialomucin) groups. We have previously shown that nematode infection also induced intestinal epithelial responses that were characterised by goblet cell hyperplasia and led to an increase in production of sialomucin, sulfomucin, Muc2, the resistin-like molecule beta (Retnlb), and St3gal4 [16–18]. Although there is a general consensus that acidic mucin plays an important role in the protection of the mucosa from infection with intestinal parasite *N. brasiliensis* in the intestine [18, 19], there are no definitive reports which specialized the kinetics of acidic mucin in bronchial epithelium after infection with *N. brasiliensis*.

Using histochemistry, immunohistochemistry, and reverse transcription real-time PCR, the present study compared the expression of goblet cell-related molecules, such as Muc2, Muc5AC, and St3gal4, in bronchial epithelial tissue from rats infected with *N. brasiliensis.* The involvement of these molecules in the sialylation of mucins and Retnlb, which is a potentially antimicrobial peptide secreted by goblet cells [20], was further investigated. 

## 2. Materials and Methods

### 2.1. Animals, Nematode Infection, and Autopsy

Specific-pathogen-free male Brown Norway/Sea (BN) rats were purchased from SLC Inc. (Shizuoka, Japan). Animals at 7 weeks of age were injected subcutaneously with 2000 *N. brasiliensis* infective-stage (L3) larvae as described previously [21]. The animals were allowed to feed *ad libitum* throughout the experiment. Uninfected animals were sacrificed as controls. Infected animals were sacrificed by loss-of-blood slaughter with low-dose ether anaesthesia at 3, 7, 10, 14, or 28 days after infection (*n* = 4 in each group). We only used low-dose ether anaesthesia to control rat's excitement at the sacrifice. After opening the thorax, the lungs were perfused with phosphate-buffered saline and a 5 × 3 × 2 mm section of right lung tissue, which included the hilus, was removed, immersed in RNA later (Ambion, Austin, TX, USA), and stored at 4°C until RNA extraction. A 5 × 10 × 2 mm section of left lung tissue including the hilus was also removed and used for histological analysis. All experiments were performed under the animal ethics guidelines of the Kyoto Prefectural University of Medicine and were approved by the animal ethics committee of Kyoto Prefectural University of Medicine.

### 2.2. Histology and Goblet Cell Count

The left lung tissue was fixed in 4% buffered formalin overnight, embedded in paraffin, and 5 *μ*m sections were cut. Goblet cells were identified with the periodic acid-Schiff (PAS) reaction with hematoxylin nuclear staining. Using a microscope, bronchi with a diameter of 200–400 *μ*m were selected, and the numbers of goblet cells and epithelial cell nuclei in each bronchus were counted. The average number of goblet cells/100 epithelial cells in five bronchi was used as the representative value in a given animal, and the means and standard error (SE) of four animals were calculated. The numbers of worms in these sections were counted under a microscope, and the numbers/mm^2^ of lung were determined. The lung sections, corresponding to an area of 120 mm^2^, were analysed per animal. The means and SE of four animals were calculated.

### 2.3. Histochemical Methods

To identify sulfomucin-positive goblet cells, the high iron diamine-alcian blue (HID-AB, pH 2.5) method was employed. Briefly, slides were immersed in HID solution for 18–24 h [22–24]. After washing in 3% acetic acid, the slides were immersed in AB solution (10 mg/mL) (pH 2.5) for 30 min. Sulfomucin stained with HID was coloured dark brown, while acidic mucin other than sulfomucin was stained blue. To identify neuraminidase-sensitive AB-positive sialomucin, sections were incubated for 90 minutes at 37°C in a solution of 0.01 M acetate buffer (pH 4.5) with or without 0.5 U/mL neuraminidase [25]. After washing with phosphate-buffered saline, slides were stained with AB solution for 30 min. HID-positive goblet cells and the numbers of neuraminidase-sensitive AB-positive goblet cells were counted using a microscope as described previosly.

### 2.4. Immunohistochemistry for Muc2 and Muc5AC

Sections of 5 *μ*m thickness were cut and mounted on poly-L-lysine-coated slides. The dewaxed sections were treated with 1% hydrogen peroxide in 0.05 M Tris-HCl for 20 min, immersed in 0.01 M sodium citrate buffer (pH 6.0), and autoclaved at 121°C for 10 min for antigen retrieval according to the method described by Bankfalvi et al. [26]. The sections were then incubated with goat anti-Muc2 IgG or goat anti-Muc5AC IgG (Santa Cruz Biotechnology Inc., CA, USA) overnight, followed by incubation with an amino acid polymer solution, which conjugated the rabbit anti-goat IgG Fab fraction and peroxidase (N-Histofine, Nichirei, Tokyo, Japan), for 30 min. The final reaction was carried out in 0.05 M Tris-HCl buffer (pH 7.4) containing 0.2 mg/mL 3,3′-diaminobenzidine tetrahydrochloride (Dojindo Lab., Kumamoto, Japan) and 0.005% hydrogen peroxide. As a negative control, normal goat IgG was employed instead of Muc2- or Muc5AC-specific antibody.

### 2.5. Extraction of Total RNA, cDNA Synthesis, Real-Time PCR, and Relative Quantification of Gene Expression

Total RNA was extracted from the right lung using TRIZOL reagent (Life Technologies, Rockville, MD, USA) in accordance with the manufacturer's instructions. Two microgram aliquots of RNA were reverse transcribed in 20 *μ*L of reverse transcription buffer containing 5 mM MgCl_2_, 1 mM dNTP mixture, 1 U/*μ*L RNase inhibitor, 0.25 U/*μ*L AMV reverse transcriptase, and 0.125 *μ*M oligo-dT-adaptor primer (Takara RNA LA PCR kit, Takara Biomedicals, Osaka, Japan) at 42°C for 50 min. One microlitre aliquots of the synthesized cDNA were mixed with Sybr Green PCR master mix (Applied Biosystems, Foster City, CA, USA) with appropriate primers and amplified using a real-time PCR system 7300 (Applied Biosystems). The sense and antisense primers used were as follows:

5′-CGGATCCAATGGAACAGTGG-3′ and 5′-TGCCACTGGTAGGATGATTG-3′ for Muc2; 5′-TGTTGCTATGACTGTCTCGT-3′ and 5′-CATCACAGTGCAGAGTCACA-3′ for Muc5AC; 5′-CTACACCTCTGCGACTTGGT-3′ and 5′-GGTTCTTGACAGCTCCCATC-3′ for St3gal4; and 5′-TTCCTTCTCTCGCTGATGGT-3′ and 5′-GCAGTGGCAAGTAGTTCCAT-3′ for Retnlb.

The specificity of each amplified product was confirmed by dissociation analyses giving a single sharp dissociation peak, the absence of amplified products without reverse transcription, and the appearance of a band of the expected size on electrophoresis of the amplified product. For the amplification of *β*-actin, Actb primers (Rn00667869, Applied Biosystems) and Taq-Man PCR master mix (Applied Biosystems) were used. For relative quantification, standard curves of the threshold cycle (Ct) of amplification of each target against the log concentration of the total RNA were created using the cDNA samples that showed the lowest Ct values in preliminary runs, and relative quantification was performed for each sample. All quantified values were normalised to those of *β*-actin (quantified value for a certain target/quantified value for *β*-actin).

### 2.6. Statistical Analysis

Student's *t*-test (two-tailed) was employed for statistical analysis; a *P* value of less than 0.05 was considered significant.

## 3. Results

### 3.1. Profiles of Goblet Cell Hyperplasia in the Bronchial Epithelium after Nematode Infection

**Figure 1:**
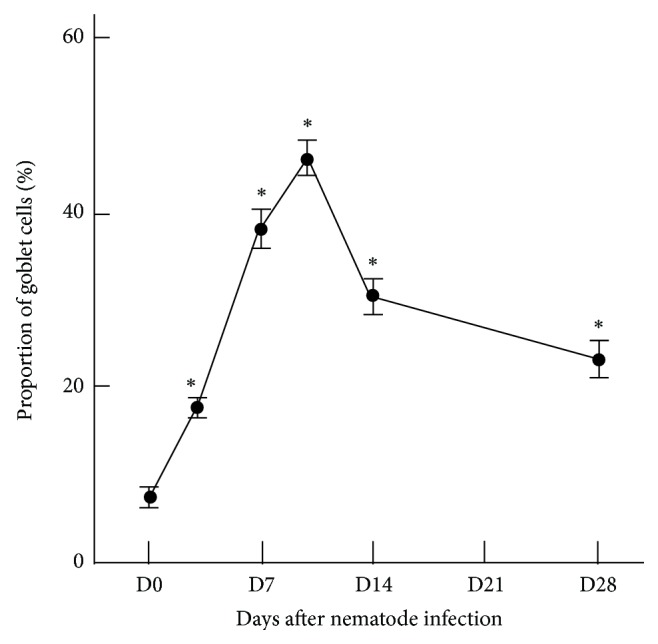
Goblet cell hyperplasia in the bronchial epithelium of BN rats after *N. brasiliensis* infection. D0–D28 represent days after primary infection. Dots and bars shown are the average numbers of goblet cells per 100 bronchial epithelium nuclei and SE. ∗Significantly different from the corresponding value for D0 (^*^ *P* < 0.01).

**Figure 2:**
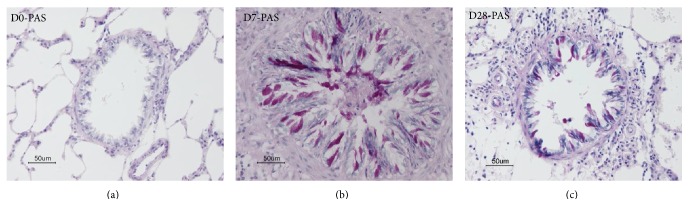
PAS staining of the rat airway epithelium in BN rats after* N. brasiliensis* infection. (a)–(c) PAS and hematoxylin staining showing marked goblet cell hyperplasia in the bronchial epithelium; (a) noninfected control; (b) 7 days after infection; (c) 28 days after infection.

Initially, *N. brasiliensis *larvae migrate to the lungs within 24–48 hours after percutaneous infection and move quickly into their final habitat, the jejunum, 3-4 days after infection where they grow into adult worms by day 7. The kinetics of airway goblet cell responses was examined in rats at 3, 7, 10, 14, or 28 days after infection. The number of goblet cells stained with PAS reaction in the bronchial epithelium increased significantly as early as 3 days after infection and peaked at 10 days after infection (Figures 1 and 2(a)–2(c)), suggesting that airway goblet cell hyperplasia proceeded even after the major number of larvae left the lungs. In the present study, 0.08 ± 0.06 larvae and 0.01 ± 0.005 larvae per 0.1 mm^2^ of lung section were found at 3 and 7 days after infection, respectively, suggesting that a small number of larvae remained in the lungs for at least 7 days after infection. Thus, although a significant number of larvae left the lungs within a few days, a small number of larvae remained.

### 3.2. Profiles of Increased Sialomucin Positive Goblet Cells in the Bronchial Epithelium after Nematode Infection

**Figure 3:**
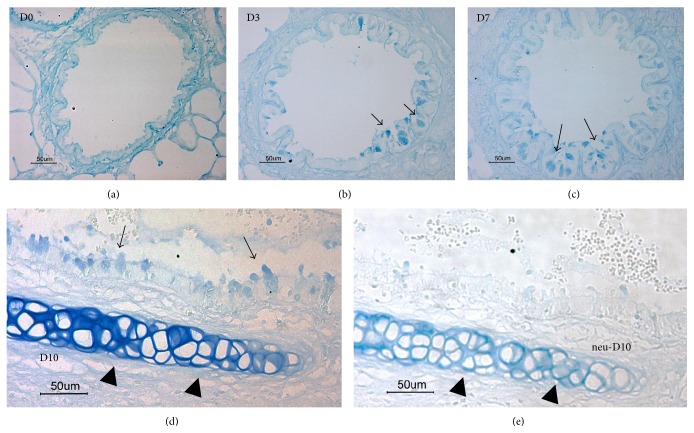
Alcian blue (AB) staining on the bronchial epithelial mucus in BN rat after *N. brasiliensis* infection. The majority of goblet cells were stained with AB after infection (arrow). Tracheal cartilages are admitted in the figure (arrowhead): (a) noninfected control; (b) 3 days after infection; (c) 7 days after infection; (d) 10 days after infection; (e) 10 days after infection, pretreated with neuraminidase.

To characterise the goblet cell mucins in bronchial epithelial tissue from rats infected with *N. brasiliensis*, the HID-AB pH 2.5 method was used. The AB staining method was employed to identify sialomucin and the HID staining method was used to identify sulfomucin. In control (noninfected) bronchial epithelium, AB-positive goblet cells were rarely found, whereas the majority of goblet cells were stained with AB after infection (Figures 3(a)–3(d)) in parallel with increases in the numbers of PAS-positive goblet cells. To determine whether AB-positive goblet cells contained sialomucin, parallel sections were cut and treated with neuraminidase before AB staining. Goblet cells in neuraminidase-treated sections failed to stain with AB (Figures 3(d) and 3(e)), indicating that these goblet cells contained sialomucin. However, sulfomucin-producing HID-positive goblet cells were not identified in the bronchi before or after nematode infection.

### 3.3. Profiles of Increased Muc5AC and Muc2 Immunoreactivity Positive Goblet Cells in the Bronchial Epithelium after Nematode Infection

**Figure 4:**
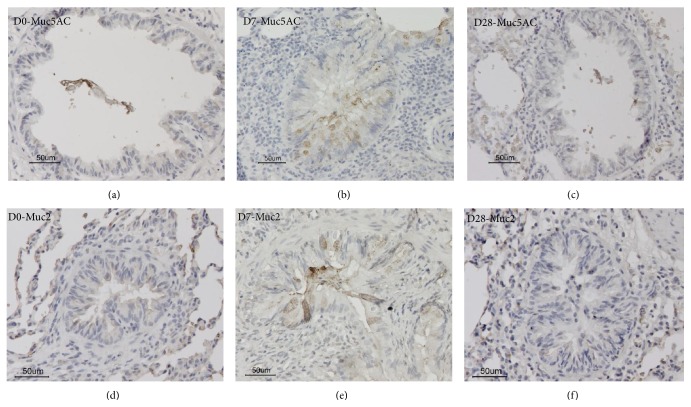
Muc immunostaining of the rat airway epithelium in BN rats after* N. brasiliensis* infection. (a)–(c) Muc5AC immunoreactivity in the bronchial epithelium; (d)–(f): Muc2 immunoreactivity in the bronchial epithelium; (a, d) noninfected control; (b, e): 7 days after infection; (c, f): 28 days after infection.

Airway mucins are pivotal to maintaining epithelial homeostasis, and mucin has been regarded as an ancient defence mechanism used by primitive organisms to create a barrier between themselves and noxious environmental stimuli. Two major secretory mucins include Muc 2 and Muc5AC. Figures 2 and 4 showed the PAS staining and Muc immunostaining of the rat airway epithelium of BN rats before, 7 days, and 28 days after *N. brasiliensis* infection.

Muc5AC is the major mucin core peptide in airway goblet cells. Using an immunohistochemical method, Muc5AC was only weakly detectable in normal bronchial goblet cells. Seven and ten days after infection, Muc5AC immunoreactivity in these goblet cells increased substantially and was associated with an increase in goblet cell number and size (Figures 4(a)–4(c)). The immunolocalisation of Muc2 in the bronchial epithelium was also examined and showed significant Muc2 immunoreactivity in goblet cells and on the bronchial cell surface 7 and 10 days after infection, but not in noninfected animals (Figures 4(a)–4(c)). These results suggested that nematode infection induced airway goblet cell hyperplasia with the production of sialomucins and immunoreactivity positive Muc5AC and Muc2 protein. 

### 3.4. Kinetics of mRNA Expression of Mucin-Related Molecular Responses in Bronchial Epithelial Cells after Nematode Infection

**Figure 5:**
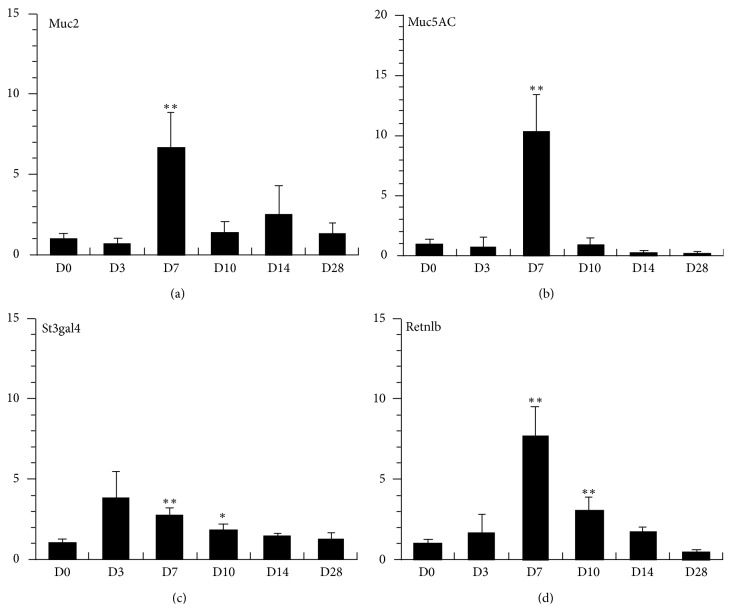
Gene transcription levels of Muc2, Muc5AC, St3gal4, and Retnlb in the lungs of BN rats after *N. brasiliensis* infection. Total RNA was extracted from the lungs, reverse transcribed, and relative quantification was carried out by real-time PCR. The quantified value for each sample was normalised with respect to that for *β*-actin. The data are mean and SE of 4 animals. The vertical axis shows the expression levels with day-0 average levels in BN rats expressed as 1.0. D0–D28 represent days after primary infection. ∗∗Significantly different from D0 values (*P* < 0.01).

Gene transcription of goblet cell-related molecules was examined by real-time PCR. Muc5AC transcription was upregulated transiently 7 days after infection in association with the development of goblet cell hyperplasia. In addition, Muc2 transcription was upregulated 7 days after infection (Figure 5). Sialyltransferase St3gal4 [27] also demonstrated a significant upregulation in transcription in the lungs 7 days after infection (Figure 5). The gene transcription of Retnlb was also examined. As shown in Figure 5, Retnlb transcription was upregulated at day 7 and at day 10 after infection.

## 4. Discussion

The present study showed that nematode infection can also induce significant goblet cell hyperplasia. The dominant airway goblet cell mucin produced after nematode infection was sialomucin, and this appeared in association with the upregulation of the mucin sialylation factor St3gal4. In this study, sialomucin-positive goblet cells in airway epithelium were upregulated after nematode infection, but sulfomucin was not. The enhanced production of sialomucin may be a response from the host to circumvent the infection, or it could be a general response mechanism to protect the mucosal membrane. We previously reported that sialomucin and sulfomucin-positive goblet cells in intestinal epithelium were upregulated after nematode infection [18]. In the intestine, the mucin forms a gel layer that covers the mucosal surface of the intestinal tract, acting as a semipermeable barrier between the lumen and epithelium. Because the great majority of microbes bind to the acidic mucin termini, acidic mucin serves as a physical barrier, preventing microbe invasion of the host epithelium [7, 28, 29]. Additionally, the sulfomucin is proposed to have evolved as a mechanism for protecting mucins from bacterial degradation [30]. Sulfomucin may be unnecessary for preventing degeneration of mucin in the lung, because respiratory tract is only the pass-through stage for *N. brasiliensis*.

In sputum, Muc5AC is the predominant oligomeric mucin, while Muc2 is almost absent [31]. The immunohistochemical experiments performed in this study demonstrated a significant increase in expression of not only Muc5AC but also Muc2 protein in goblet cells after nematode infection. Production of both types of mucins was supported by the upregulation of Muc5AC and Muc2 gene transcription. Previously, the expression of Muc2 in the airway was reported following stimulation by endotoxin from Gram-negative bacteria such as *Pseudomonas aeruginosa* [13, 14]. Similarly, the aberrant expression of Muc2 in the bronchial epithelium might have been triggered by endotoxin-like molecules and/or other unknown factors from the nematode.

Retnlb is a goblet cell-specific, nonmucin secretary peptide, which is expressed in the intestine and is likely to have a role in the defence against nematode infections in this region [20]. In the airway epithelium, Retnlb was reported to be rapidly induced in asthma mouse models [32] and in mice infected with *N. brasiliensis* [33]. In the present study, the gene expression of Retnlb in the bronchial epithelium showed transient upregulation after nematode infection in parallel with goblet cell hyperplasia, suggesting that Retnlb could play a role in protection against nematode infection, as previously reported in the intestinal epithelium [18].

## 5. Conclusions

Taken together, it was shown that nematode infection induces airway goblet cell hyperplasia, which is characterised by the overproduction of Muc2 and Muc5AC core peptides and of the nonmucin secretory peptide Retnlb. These findings were consistent with those in the intestinal goblet cell response to nematode infection, in which upregulation of Muc2 and Retnlb also occurred [16–18]. However, in contrast to the intestine, upregulation of sulfomucin-positive goblet cells was not observed in the lung. Sulfomucin may be unnecessary for preventing degeneration of mucin in the lung, because respiratory tract is only the pass-through stage for *N. brasiliensis*. These airway responses may have evolved as a result of host-parasite interactions that are beneficial for mucosal protection against infectious agents.

## Abbreviations

AB: Alcian blue
HID: High iron diamine
Muc: Mucin core peptide
Retnlb: Resistin-like molecule beta.
